# Characterizing the degeneration of nuclear membrane and mitochondria of adipose‐derived mesenchymal stem cells from patients with type II diabetes

**DOI:** 10.1111/jcmm.16484

**Published:** 2021-03-23

**Authors:** Michiko Horiguchi, Shinichi Hata, Yuya Tsurudome, Kentaro Ushijima

**Affiliations:** ^1^ Division of Pharmaceutics Faculty of Pharmaceutical Sciences Sanyo‐Onoda City University Sanyoonoda Japan; ^2^ Department of Applied Chemistry Faculty of Engineering Sanyo‐Onoda City University Sanyoonoda Japan

**Keywords:** adipose‐derived mesenchymal stem cells, mitochondria, nuclear membrane, stem cell, transmission electron microscopy, type II diabetes

## Abstract

Regenerative therapeutic approaches involving the transplantation of stem cells differentiated into insulin‐producing cells are being studied in patients with rapidly progressing severe diabetes. Adipose‐derived mesenchymal stem cells have been reported to have varied cellular characteristics depending on the biological environment of the location from which they were harvested. However, the characteristics of mesenchymal stem cells in type II diabetes have not been clarified. In this study, we observed the organelles of mesenchymal stem cells from patients with type II diabetes under a transmission electron microscope to determine the structure of stem cells in type II diabetes. Transmission electron microscopic observation of mesenchymal stem cells from healthy volunteers (N‐ADSC) and those from patients with type II diabetes (T2DM‐ADSC) revealed enlarged nuclei and degenerated mitochondrial cristae in T2DM‐ADSCs. Moreover, T2DM‐ADSCs were shown to exhibit a lower expression of Emerin, a constituent protein of the nuclear membrane, and a decreased level of mitochondrial enzyme activity. In this study, we successfully demonstrated the altered structure of nuclear membrane and the decreased mitochondrial enzyme activity in adipose‐derived mesenchymal cells from patients with type II diabetes. These findings have contributed to the understanding of type II diabetes‐associated changes in mesenchymal stem cells used for regenerative therapy.

## 1 INTRODUCTION

Type II diabetes is a lifestyle‐related disease in which patients exhibit hyperglycaemia and insulin resistance.^1^ Prolonged hyperglycaemia can lead to serious complications, such as blindness due to retinopathy, renal failure, and necrosis of the extremities.^1^ Worldwide, the total number of patients with diabetes was reportedly 463 million as of 2019 by the 9th Edition of IDF Diabetes Atlas. Current treatments for type II diabetes combine diet therapy, exercise therapy, and pharmacological therapy with hypoglycaemic agents and insulin.^2^

Meanwhile, a regenerative therapeutic approach of transplanting insulin‐producing cells is being studied in patients with rapidly progressive severe diabetes.^3^ Specifically, the transplantation of insulin‐producing cells obtained through the differentiation of autologous adipose‐derived mesenchymal stem cells or pluripotent cells is being investigated.^4^, ^5^

While mesenchymal stem cells can be isolated from various tissues, adipose‐derived mesenchymal stem cells (Adipose Delivered Stromal Cells:ADSCs) and bone marrow‐derived mesenchymal stem cells (Bone marrow Stromal Cells:BMSCs) are commonly used for regenerative therapy. While BMSCs account for only 0.01% of the cells in the bone marrow, the percentage of ADSCs in adipose tissue is approximately 500‐fold higher.^6^ Moreover, while only a limited amount of BMSCs can be harvested from the bone marrow fluid, a large amount of ADSCs can be collected from the adipose tissue in the whole body.^7^ Furthermore, ADSCs have been reported to produce more growth factors that contribute to organ repair, such as hepatocyte growth factor and vascular endothelial growth factor, than BMSCs.^7^ While BMSC proliferation has been reported to decelerate with ageing, even ADSCs from the adipose tissue of an elderly individual can easily proliferate.^8^

Although adipose‐derived mesenchymal stem cells are an excellent material for regenerative therapy, they have been shown to have different characteristics depending on the biological environment of the location from which they were harvested. Factors reported to have effects on characteristics of stem cells include the presence of underlying disease,^9^ age,^10^ race^11^ gender.^12^ However, the characteristics of stem cells in the presence of different underlying diseases remain considerably unclear. Nevertheless, a recent study has suggested that the glucose concentration in culture may affect the stem cell characteristics.^13^ However, no previous studies have clarified the characteristics of mesenchymal stem cells during type II diabetes.

In this study, we observed the organelles of mesenchymal stem cells from patients with type II diabetes under a transmission electron microscope to determine whether the characteristics of stem cells from healthy individuals differ from those obtained from patients with type II diabetes.

## 2 MATERIALS AND METHODS

### 2.1 Materials

Type II diabetes mellitus adipose‐derived stem cells (T2DM‐ADSC); Lonza, NC, USA
Normal adipose‐derived stem cells (N‐ADSC); Lonza, NC, USA
ADSC Apidose‐Derived Stem Cells Growth Medium BulletKit™; Lonza, NC, USA
8well Cell Culture slides; SPL Life Sciences, Gyeonggi‐do, Korea
ProLong™ Gold Antifade Mountant with DAPI; Invitrogen, OR, USA
60 × 15 mm Tissue culture dish; FALCON, NJ, USA
Glutaraldehyde; EM grade, Electron Microscopy Science. PA., USA
Osmium tetra‐oxide; Crystal, Heraeus Chemicals South Africa, South Africa
Ethanol; Wako, Osaka, Japan
Toluidine bleu: Wako, Osaka, Japan
Grid: Cu 200 mesh, EM fine grid, Nisshinn EM, Tokyo, Japan
Epoxy resin: TAAB Laboratories, UK
Lead staining solution; A stable lead by modification of Sato's method.
Anti‐Emerin antibody ab40688; abcam, OR, USA
Alexa Fluor 488 goat anti‐rabbit IgG(H + L), PROTEINTECH, IL, USA
Anti‐Lamin A/C antibody 2032; Cell Signaling Technology, MA, USA
HRP‐labelled secondary antibody; Santa Cruz Biotechnology, TX, USA
MTT Cell Proliferation and Cytotoxicity Assay Kit; Boster, CA., USA
Human mitochondrial DNA (mt DNA) monitoring primer 7246; TAKARA, Shiga, Japan
SYBR^®^ Premix Ex Taq™ II (Tli RNaseH Plus); TAKARA, Shiga, Japan

### 2.2 Culture conditions for mesenchymal stem cells from healthy volunteers (N‐ADSC) and patients with type II diabetes (T2DM‐ADSC)

N‐ADSCs or T2DM‐ADSCs were cultured in 10‐cm petri dishes containing 12 mL of a dedicated medium in a 5% CO_2_ incubator at 37°C. When the cells were 70%–80% confluent, they were seeded in a new dish at a density of approximately 5000 cells/cm^2^ and maintained. A trypsin/EDTA solution was used to detach the cells.

Two ADSC lines used in this study were purchased from LONZA; these were primary culture products guaranteed to be ≥90% positive for CD13, CD29, CD44, CD73, CD90, CD105, and CD166 and ≤5% negative for CD14, CD31, and CD45. The medium used was a high‐glucose special medium containing serum, antibiotics, and growth factors, which was purchased from LONZA. ADSC used in experiments were those with a passage number between 2 and 5 and that confirmed the expression of stem cell markers.

N‐ADSC and T2DM‐ADSC purchased from LONZA for use in this study were established from donors with legal permission and informed consent.

### 2.3 Observation of cells with a phase‐contrast microscope

N‐ADSCs or T2DM‐ADSCs were seeded on an 8‐well culture slide at a density of 5000 cells/cm^2^. After 48 hours, they were fixed with 4% paraformaldehyde for 20 minutes at room temperature. After washing with T‐TBS, the cells were mounted with a mounting medium containing DAPI. They were observed with an inverted fluorescence phase‐contrast microscope (Keyence, BZ‐X700).

### 2.4 Observation of cells with a transmission electron microscope

N‐ADSCs or T2DM‐ADSCs were seeded in a 60 × 15‐mm tissue culture dish at a density of 5000 cells/cm^2^. The cells were observed with a transmission electron microscope as per normal procedures.

The samples of cells for TEM were fixed in phosphate buffered 2% glutaraldehyde, and subsequently post‐fixed in 2% osmium tetra‐oxide for 2 hours in the ice bath. Then, the specimens were dehydrated in a graded ethanol and embedded in the epoxy resin. Ultrathin sections were obtained by ultramicrotome technique. Ultrathin sections stained with uranyl acetate for 15 minutes and lead staining solution for 5 minutes were submitted to TEM observation (HITACHI, H‐7600).

### 2.5 Immunostaining of Emerin, Lamin A/C and DAPI and fluorescence microscopy

N‐ADSCs or T2DM‐ADSCs were seeded on an 8‐well culture slide at a density of 5000 cells/cm^2^. After 48 hours, the cells were fixed with 4% paraformaldehyde for 15 minutes at room temperature. Additionally, they were fixed with 100% methanol for 5 minutes at room temperature. After the addition of T‐TBS, the slide was allowed to stand at room temperature for 20 minutes. After blocking with 10% BSA, the fixed cells underwent a primary antibody reaction with 1000‐fold diluted Emerin antibody and Lamin A/C antibody at room temperature for 1 hour. After washing, the cells underwent a secondary antibody reaction with 1000‐fold diluted Alexa Fluor^®^ 488 antibody at room temperature for 1 hour. After washing, the cells were mounted with a mounting medium containing DAPI. Emerin and DAPI were observed with an inverted fluorescence phase‐contrast microscope (Keyence, BZ‐X700). For the fluorescence intensity‐based quantification, we used Fiji ImageJ.

### 2.6 Quantification of the protein expression levels by the Western blot

N‐ADSC and T2DM‐ADSC were seeded in 10‐cm petri dishes at an approximate density of 5000 cells/cm^2^. After isolating the nuclear fraction, proteins were extracted from it. Samples were subjected to SDS‐PAGE after adjusting the loading amount per lane/sample by protein concentration. Proteins were then transferred to the PVDF membrane. The membrane was blocked with 5% BSA and incubated with 1/1000 dilution of anti‐Emerin antibody and Lamin A/C antibody at 4°C overnight. Following washing, the membrane was further incubated with HRP‐labelled secondary antibody for 1 hour at room temperature. Finally, it was developed with chemiluminescence reagent to visualize the target protein.

### 2.7 Evaluation of mitochondrial enzyme activity with MTT assay

N‐ADSCs or T2DM‐ADSCs were seeded on a 96‐well plate at a density of 2000 cells/well. An MTT labelling reagent (10 μL) was added to each well. The plate was incubated at 37°C for 4 hours. After adding the MTT elution solution and mixing the resulting solution well, the plate was allowed to stand in an incubator at 37°C for 6 hours. For quantification, the absorbance of the purple dye at a wavelength of 570 nm was measured with a light absorption metre.

### 2.8 Quantified mitochondrial DNA copy number by PCR

N‐ADSC and T2DM‐ADSC were seeded in 10‐cm petri dishes at an approximate density of 5000 cells/cm^2^. The template DNA was obtained from the cell lysate using NucleoSpin^®^ Tissue. The mitochondrial DNA copy numbers were quantified using Human mitochondrial DNA (mt DNA) monitoring primer set and SYBR^®^ Premix Ex Taq™ II (Tli RNaseH Plus). In quantitative PCR, a cycle of amplification at 95°C for 5 seconds and 60°C for 60 seconds was repeated 40 times on a quantitative PCR device StepOne‐Plus‐01. The mitochondrial DNA, ND1 and ND2 copy numbers were determined using SLCO2B1 and SERPINA1 as the internal standard.

## 3 RESULTS

This study clarified the structural differences of mesenchymal stem cells from patients with type II diabetes when compared with those obtained from healthy individuals. We focused on adipose‐derived mesenchymal stem cells, which are a promising regenerative therapeutic material for severe diabetes. Mesenchymal stem cells from healthy volunteers (N‐ADSC) and those from patients with type II diabetes (T2DM‐ADSC) have been verified to be positive for the following stem cell markers: CD13, CD29, CD44, CD73, CD90, CD105, and CD166. They are also known to be negative for CD14, CD31, and CD45.

**FIGURE 1:**
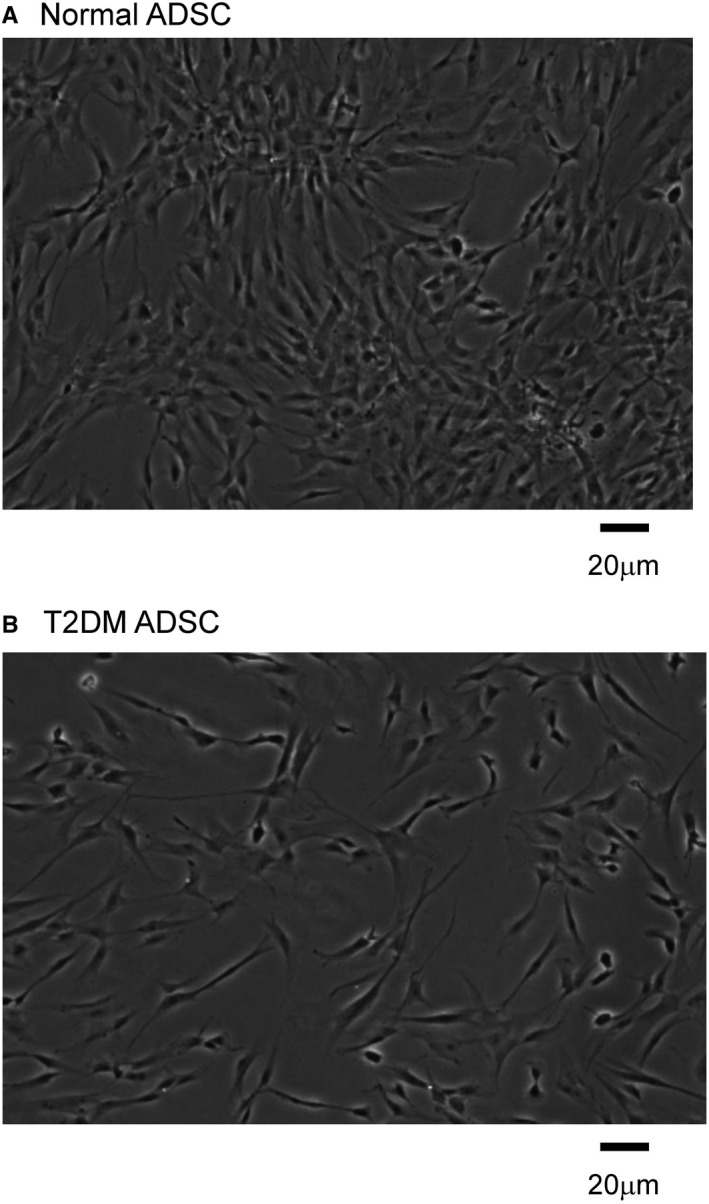
Observation of the shape and density of adipose‐derived mesenchymal stem cells. The shape and density of human adipose‐derived mesenchymal stem cells cultured and fixed with paraformaldehyde were observed with an inverted phase‐contrast microscope. The figure shows mesenchymal stem cells from healthy volunteers (A) and patients with type II diabetes (B). The scale bar denotes 20 μm

First, N‐ADSCs and T2DM‐ADSCs were observed with an inverted fluorescence phase‐contrast microscope (Keyence, BZ‐X700). Results showed that T2DM‐ADSCs were more flat and larger with swollen cytoplasm than N‐ADSCs (Figure 1). Furthermore, T2DM‐ADSCs were more heterogeneous and had a lower cell density (Figure 1).

**FIGURE 2:**
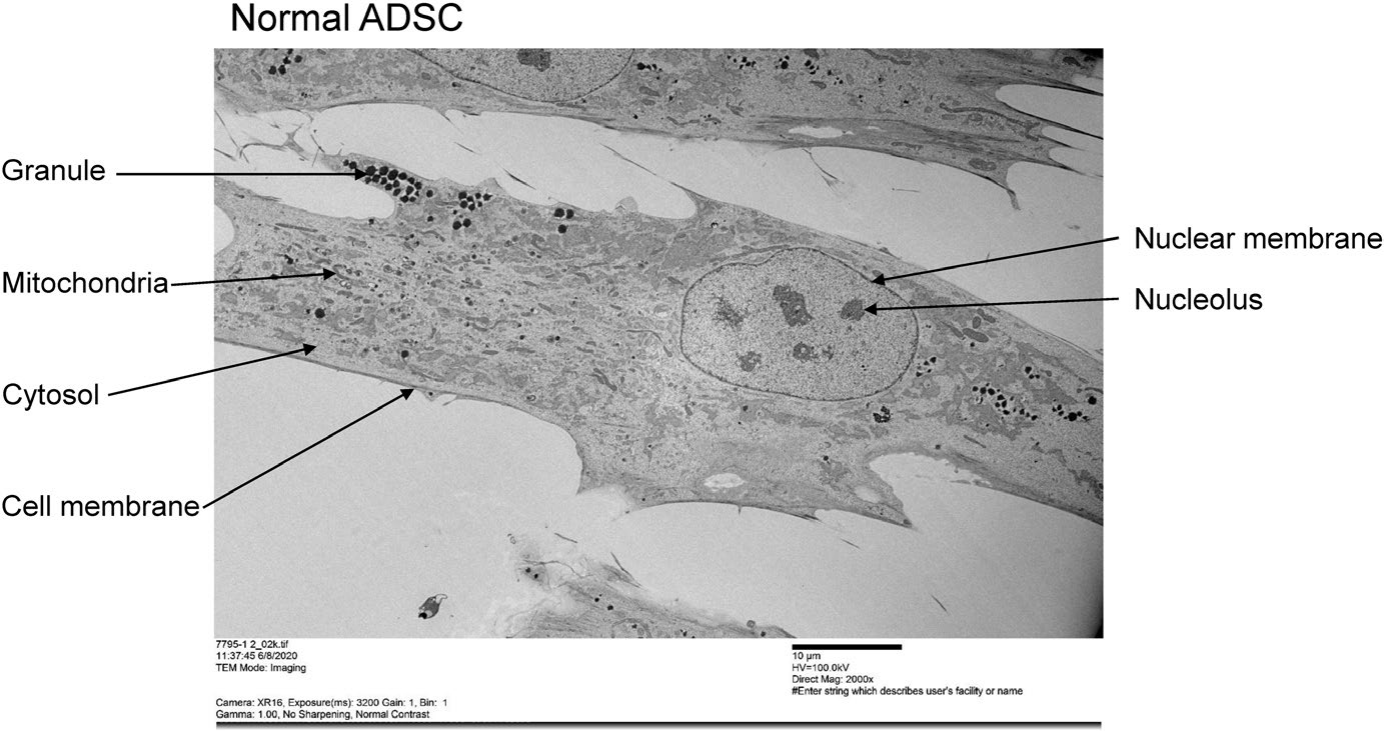
Observation of the overview of mesenchymal stem cells from healthy volunteers. A transmission electron microscope (TEM) was used to observe the overview of mesenchymal stem cells from healthy volunteers. The scale bar denotes 10 μm

**FIGURE 3:**
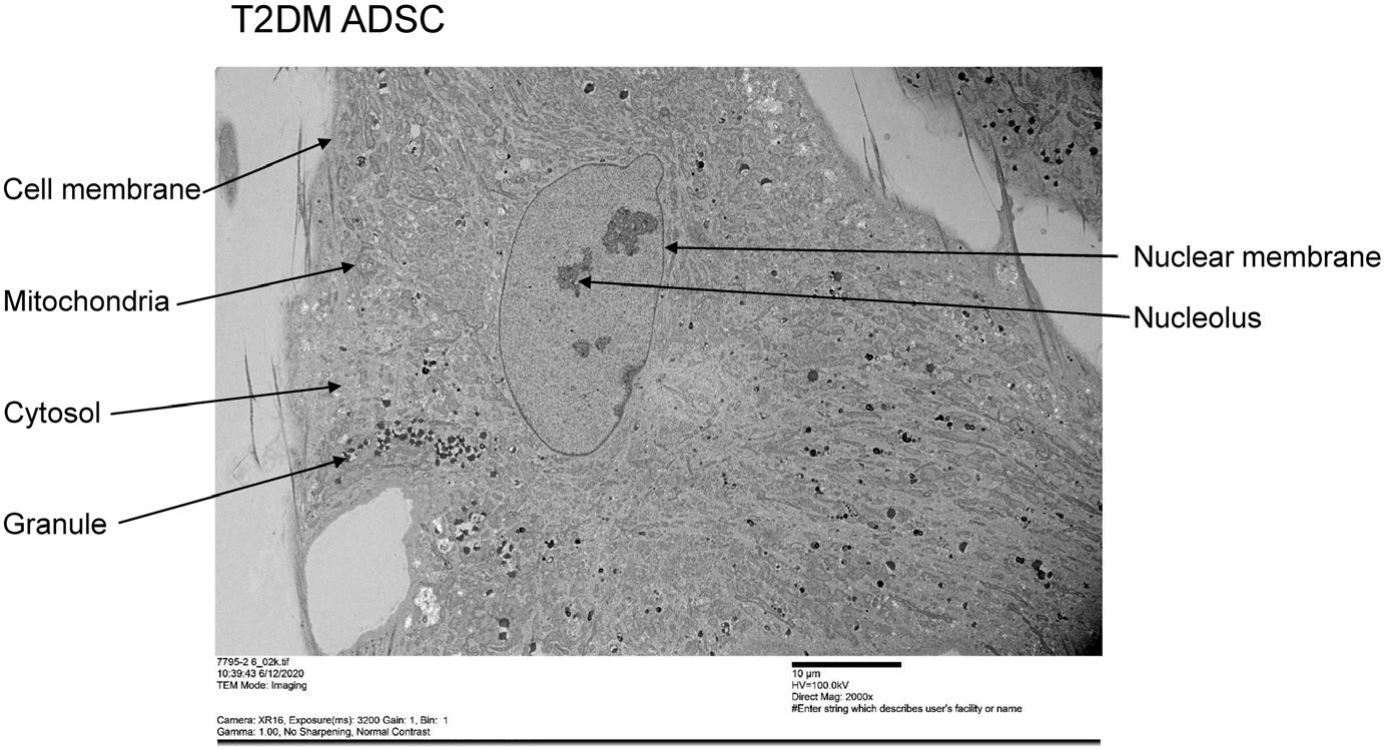
Observation of the overview of mesenchymal stem cells from patients with type II diabetes. A transmission electron microscope (TEM) was used to observe the overview of mesenchymal stem cells from patients with type II diabetes. The scale bar denotes 10 μm

Next, each cell in the preparations of N‐ADSCs and T2DM‐ADSCs was observed with a transmission electron microscope (Hitachi, H‐7600). The T2DM‐ADSCs were found to be more enlarged; besides, they had a larger number of vesicles and a more swollen nucleus than the N‐ADSCs (Figures 2 and 3). Therefore, the organelles were observed in detail with a focus on the nucleus and nuclear membrane.

The nuclear membrane has a lipid bilayer structure comprising an inner membrane and an outer membrane, which is continuous with the endoplasmic reticulum. The pores of the nuclear membrane are composed of nuclear pore complexes formed by many proteins, serving as pathways allowing substances to move between the inside and outside of the nucleus. The space between the inner and outer membranes is referred to as the perinuclear space with ~20‐40‐nm width. Inside the inner membrane, nuclear laminae, intermediate filaments comprising lamins, form a lattice‐shaped lining structure that maintains the shape of the nucleus.

**FIGURE 4:**
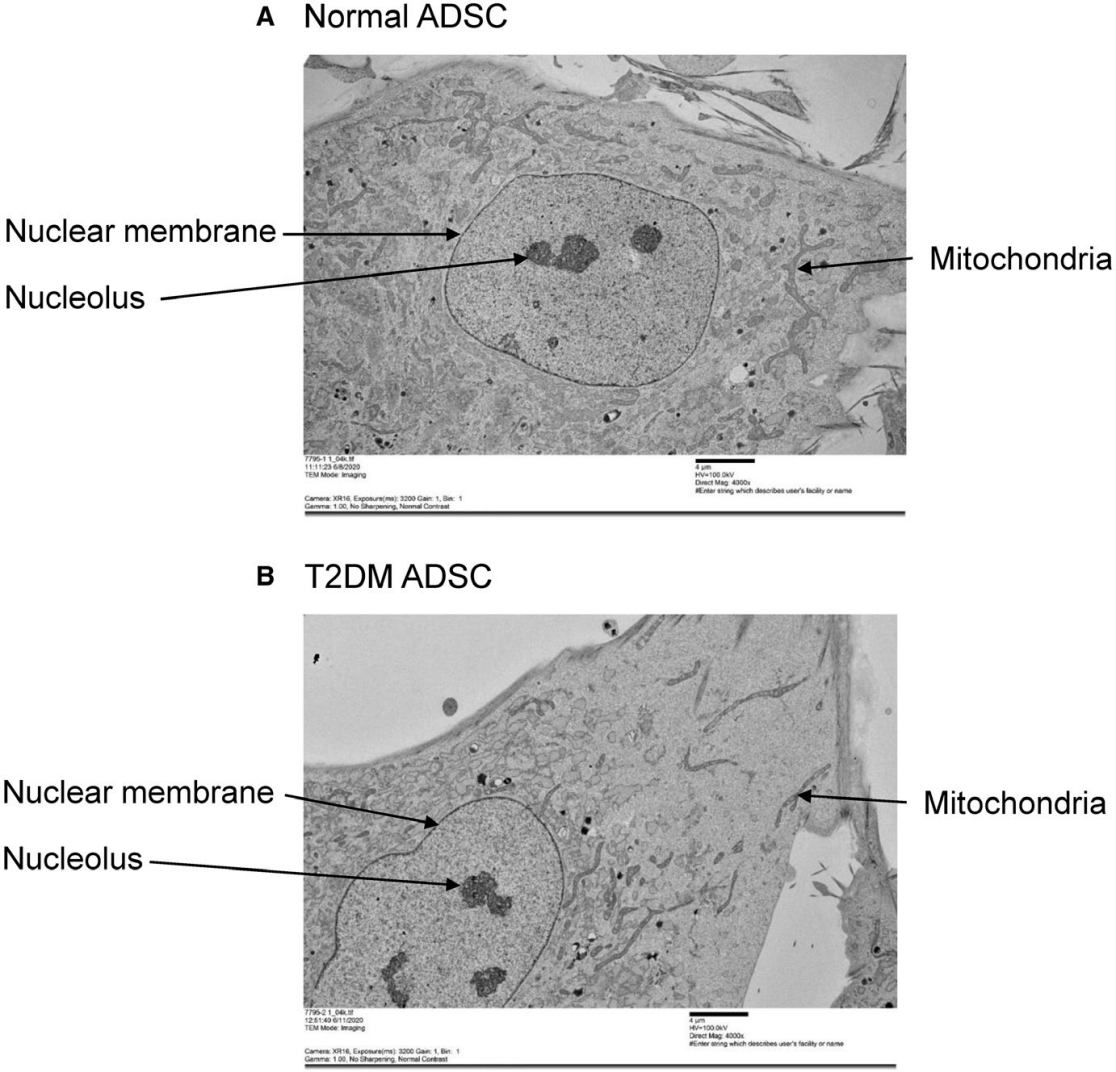
Observation of the nucleus and nuclear envelope of mesenchymal stem cells. A transmission electron microscope (TEM) was used to observe the nuclei and nuclear membranes of mesenchymal stem cells from healthy volunteers (A) and patients with type II diabetes (B). The scale bar denotes 4 μm

**FIGURE 5:**
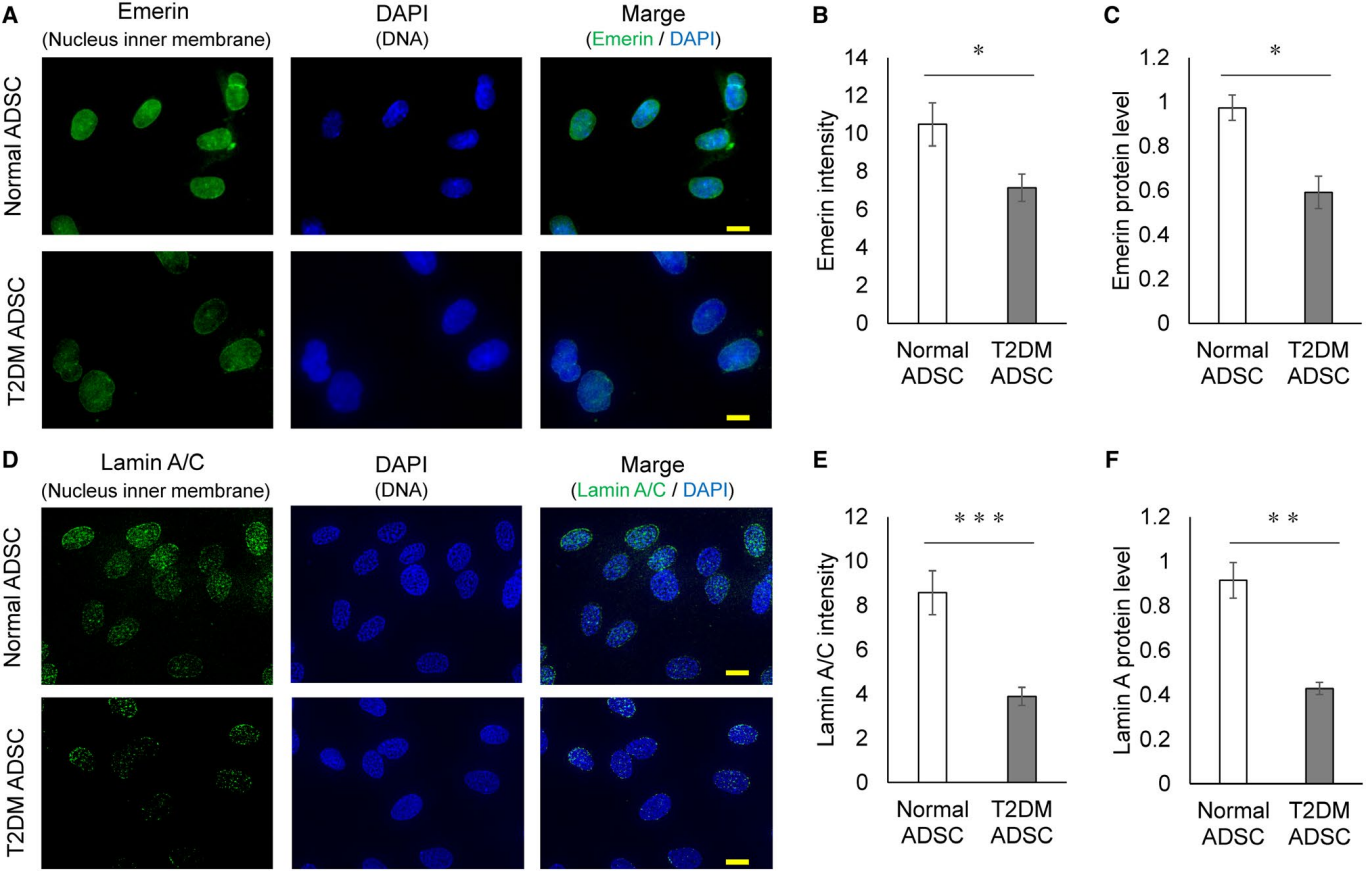
Observation of the nuclear membrane protein in mesenchymal stem cells. (A) Mesenchymal stem cells from healthy volunteers and patients with type II diabetes were stained for the nuclear membrane protein Emerin (green) and with 4′,6‐diamidino‐2‐phenylindole (DAPI) that binds to the DNA (blue). The scale bar denotes 10 μm. (B) The fluorescence intensity of Emerin immunostaining performed using Image J. The fluorescence intensity of Emerin was shown in the form of average ± SEM. The number of samples was n = 10. The fluorescence intensity of Emerin immunostaining was evaluated two times independently. The *t*‐test was used to test for statistical significance. *P* = 0.0232. (C) The protein levels of Emerin quantified by the Western blot. The protein levels of Emerin were shown in the form of average ± SEM. The number of samples was n = 3. The protein levels of Emerin were evaluated three times independently. The *t*‐test was used to test for statistical significance. *P* = 0.0146. (D) Mesenchymal stem cells from healthy volunteers and patients with type II diabetes were stained for the nuclear membrane protein Lamin A/C (green) and DAPI (blue). The scale bar denotes 10 μm. (E) The fluorescence intensity of Lamin A/C immunostaining performed using Image J. The fluorescence intensity of Lamin A/C was shown in the form of average ± SEM. The number of samples was n = 10. The fluorescence intensity of Lamin A/C immunostaining was evaluated two times independently. The *t*‐test was used to test for statistical significance. *P* = 0.0004. (F) The protein levels of Lamin A/C quantified by the Western blot. The protein levels of Lamin A/C were shown in the form of average ± SEM. The number of samples was n = 3. The protein levels of Lamin A/C were evaluated three times independently. The t‐test was used to test for statistical significance. *P* = 0.0045

While the nuclear membrane was not deformed in the N‐ADSCs (Figure 4A), it was deformed and swollen in the T2DM‐ADSCs. We then focused on the nuclear protein Emerin and compared its expression levels with immunostaining. Emerin is a protein found in the inner nuclear membrane. Emerin was detected by the fluorescent dye fluorescein isothiocyanate (FITC) (green), while the DNA was stained with 4′,6‐diamidino‐2‐phenylindole (DAPI) (blue) (Figure 5A). The results of fluorescence intensity‐based quantification showed a significant decrease in the Emerin fluorescence intensity in T2DM‐ADSC compared with that in N‐ADSC (Figure 5B). The expression level of the Emerin protein in T2DM‐ADSC quantified by the Western blot was significantly lower than that in N‐ADSC (Figure 5C).

We focused on Lamin A, a fibrous protein responsible for transcriptional regulation in the cell nucleus and maintenance of the nuclear membrane structure. Lamin A/C was immunostained with anti‐Lamin A/C antibody and the secondary antibody labelled with the green fluorescent dye FITC. DNA was counterstained with blue fluorescent dye DAPI (Figure 5D). Quantification based on the fluorescence intensity showed that the Lamin A/C expression in T2DM‐ADSC was significantly lower than that in N‐ADSC (Figure 5E). Furthermore, the result of quantification of the Lamin A protein by the Western blot revealed that the expression level of the Lamin A protein in T2DM‐ADSC was significantly lower than that in N‐ADSC (Figure 5F). Thus, our results showed that there was reduced expression of a protein constituting the nuclear membrane and enlargement of the nucleus in T2DM‐ADSCs.

**FIGURE 6:**
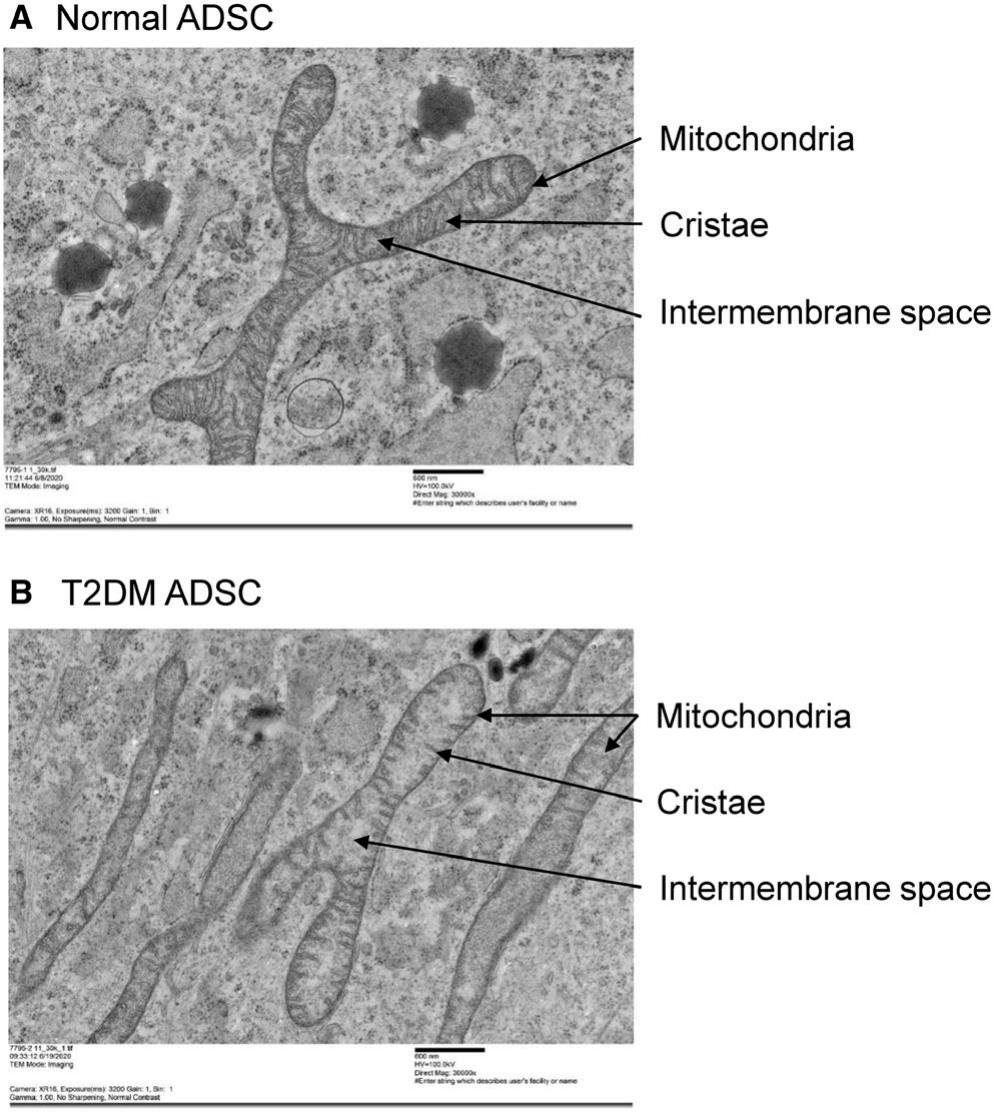
Observation of mitochondria in mesenchymal stem cells. A transmission electron microscope (TEM) was used to observe the mitochondria in mesenchymal stem cells from healthy volunteers (A) and patients with type II diabetes (B). The scale bar denotes 600 nm

**FIGURE 7:**
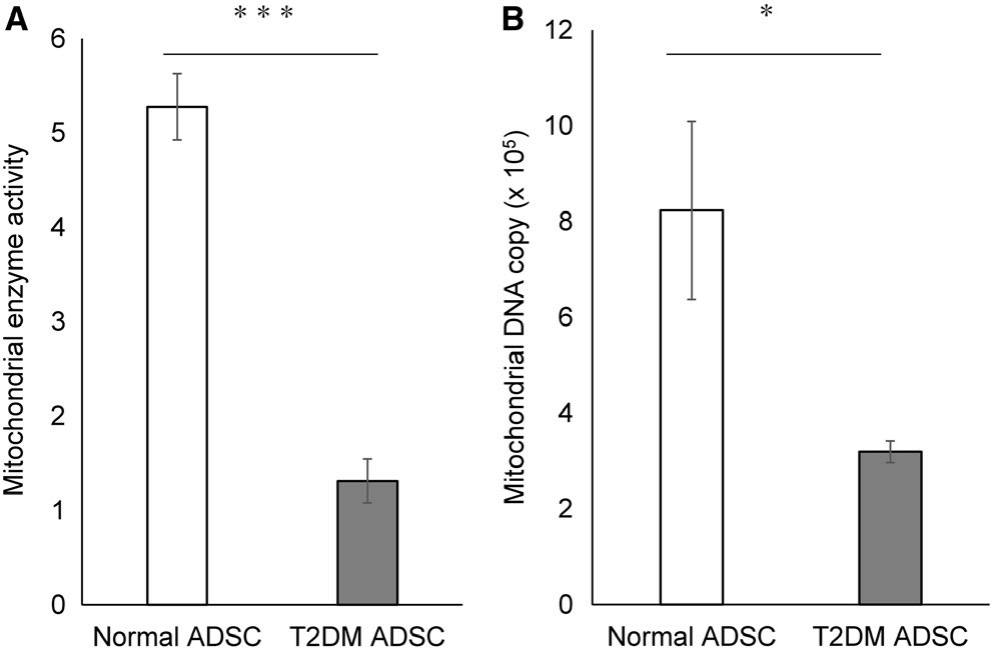
Evaluation of the mitochondrial enzyme activity. (A) The MTT assay was used to quantify the mitochondrial enzyme activity. In the graph, white and grey columns denote the mitochondrial enzyme activity in the mesenchymal stem cells from healthy volunteers and patients with type II diabetes, respectively. The activity was shown in the form of average ± SEM. The number of samples was n = 9. The activity was evaluated three times independently. The t‐test was used to test for statistical significance. *P* = 0.0007. (B) The mitochondrial DNA copy numbers were quantified using human mitochondrial DNA (mt DNA) monitoring primer set. The mitochondrial DNA copy numbers were shown in the form of average ± SEM. The number of samples was n = 5. The mitochondrial DNA copy numbers were evaluated three times independently. The *t*‐test was used to test for statistical significance. *P* = 0.0425

We performed transmission electron microscopic observations to determine whether the mitochondria, which are responsible for energy production, undergo any changes in association with the enlargement of the cell and the nuclear membranes. The mitochondrion is an organelle with a dual membrane structure encapsulated by two lipid membranes. The inside of the inner membrane is referred to as the matrix and the inner membrane is invaginated into the matrix to form plate‐shaped cristae structures. In the inner mitochondrial membrane, respiratory chain complexes and adenosine triphosphate (ATP) synthase produce the biological energy source, ATP. In N‐ADSCs, mitochondrial cristae were observed as regularly overlapping folds (Figure 6A). In contrast, the mitochondria in T2DM‐ADSCs were swollen and had shorter cristae with a smaller number of folds that no longer overlapped with one another (Figure 6B). Subsequently, we evaluated the activity of mitochondrial enzymes using MTT (3‐(4,5‐di‐methylthiazol‐2‐yl)‐2,5‐diphenyltetrazolium bromide) assays to compare the mitochondrial function. In living cells, MTT is reduced to purple‐coloured formazan mainly by the mitochondrial reductases. In MTT assays of N‐ADSCs and T2DM‐ADSCs, the absorbance values were 5.28 ± 0.61 and 1.31 ± 0.40, respectively, showing that the mitochondrial enzyme activity in T2DM‐ADSCs was significantly decreased to a fourth of that in N‐ADSCs (Figure 7A). Quantification of mitochondrial DNA copy number is useful for monitoring mitochondria. ND1 and ND2, which are mitochondrial‐specific DNAs, were measured by quantitative PCR. In T2DM‐ADSC, the number of mitochondrial DNA copies was significantly reduced compared with Normal‐ADSC (Figure 7B). These results revealed degenerated cristae and reduced enzyme activity in the mitochondria of T2DM‐ADSCs.

## 4 DISCUSSION

A regenerative therapeutic approach with insulin‐producing cells derived from stem cells has been investigated for the treatment of severe type II diabetes.^14^, ^15^ In the regenerative therapy for type II diabetes, pluripotent stem cells, such as autologous adipose‐derived mesenchymal stem cells, bone marrow‐derived mesenchymal stem cells, and iPS cells have been studied.^16^, ^17^, ^18^, ^19^, ^20^ However, no studies have investigated whether stem cells from diabetic patients have the same cell structure as the ones sampled from healthy individuals.

Summarizing the present data, we successfully clarified the cellular characteristics of adipose‐derived mesenchymal stem cells in patients with type II diabetes. T2DM‐ADSCs were shown to have large‐sized, heterogeneous flat cells with swollen cytoplasm when compared with N‐ADSCs (Figures 1, 2, 3). T2DM‐ADSCs showed deformation and swelling of the nuclear membrane (Figure 4) and a decreased expression level of Emerin, a membrane protein found in the inner nuclear membrane (Figure 5). Moreover, we demonstrated denatured mitochondrial cristae (Figure 6) and decreased enzyme activity (Figure 7) in T2DM‐ADSCs. This study provided novel findings, indicating that mesenchymal stem cells in patients with type II diabetes undergo structural changes.

We used a transmission electron microscope to observe organelles and found degeneration only in nuclear membranes and mitochondria. In contrast, by transmission electron microscope, no changes were detected in other organelles, such as Golgi apparatus, endoplasmic reticulum, and nucleolus. The degeneration of nuclear membranes and mitochondria results in gene damage and defective energy metabolism. The observed degeneration of nuclear membranes and mitochondria may result in functional deterioration of stem cells in patients with diabetes.

The donor age has been reported to be associated with deteriorated mitochondrial function in mesenchymal stem cells.^10^, ^12^, ^21^, ^22^ Mesenchymal stem cells evaluated in this study were obtained from the adipose tissue of Caucasian female donors with a mean age of 31 years (range 23‐39 years). Therefore, differences in age, race, and sex are unlikely to have effects on the results. Accordingly, the results of this study can be concluded to suggest associations of the history of type 2 diabetes with the nuclear membrane and mitochondrial functions.

In this study, the nuclear membrane of T2DM‐ADSCs was shown to be deformed and swollen. In the cellular nucleus, the intermediate filament protein, lamin, forms a network structure called lamina,^23^ which connects the nuclear membrane with chromatin. The laminae stabilize the nuclear structure and are involved in chromatin organization, gene transcription, and DNA replication.^24^ In this study, the expression level of Emerin, a membrane protein found in the inner nuclear membrane, was found to be decreased in T2DM‐ADSCs (Figure 5). Emerin, a member of the nuclear lamina‐associated protein family, is a serine‐rich nuclear membrane protein.^25^ The deformation and swelling of the nuclear membrane and Emerin decrease observed in T2DM‐ADSCs may affect chromatin structure degeneration and gene transcription.

In this study, the mitochondrial cristae in T2DM‐ADSC were found to be degenerated. The mitochondria have been reported to migrate actively in cells and undergo fusion and fission repeatedly. GTPases are involved in these processes; mitofusion (Mfn) 1 and Mfn2 in the outer mitochondrial membrane cooperatively control the fusion of the outer membrane, and optic atrophy type 1 (OPA1) controls the formation of the inner membrane and cristae.^26^, ^27^ Structural changes in mitochondria alter the balanced production of the biological energy source, ATP, which greatly affects the homeostasis of cellular metabolism. In this study, the mitochondrial enzyme activity was found to be decreased in T2DM‐ADSCs, which in turn affects the ATP production capacity of mitochondria through oxidative phosphorylation, resulting in alteration of energy metabolism. The results suggest that the evaluation of mitochondrial function is important in understanding the structural characteristics of mesenchymal stem cells from diabetic patients for use in regenerative therapy.

Abnormal mitochondria with functional insufficiency are eliminated by mitochondrial autophagy (mitophagy), a quality control mechanism of the mitochondria.^28^ Mitophagy has been reported to contribute to the homeostasis of intracellular mitochondria through the degradation of mitochondria.^29^ Mitophagy is a type of autophagy that maintains the homeostasis of intracellular proteins and organelles.^30^ In mitophagy, the mitochondria are selectively sequestered in autophagosomes and then degraded by lysosomes.^30^ In this study, a greater number of cellular vesicles were observed in T2DM‐ADSC than in N‐ADSC (Figure 3). However, electron microscopic data did not reveal whether mitophagy was occurring in the observed vesicles. In future, we plan to analyse the mitophagy in T2DM‐ADSCs.

In this study, we focused on the structures of organelles in adipose‐derived mesenchymal stem cells and demonstrated the degeneration of nuclear membranes and mitochondria in T2DM‐ADSC. However, the underlying molecular mechanism was not determined. In T2DM, neovascularization, secretion of proinflammatory cytokines, increased oxidative stress markers, and decreased differentiation/growth factors have been shown to affect functional deterioration of mesenchymal stem cells.^31^, ^32^ Multiple studies have reported that ADSC senescence is caused by oxidative stress in mitochondria and the activation of the Raf1/NF‐κB signalling pathway and is associated with abnormalities in tissue repair and immune regulatory and other functions.^33^, ^34^ Moving forward, we hope to analyse the detailed molecular mechanisms underlying the effects of degeneration of nuclear membranes and mitochondria on mesenchymal stem cell function.

Recently, the clinical research results and treatment outcomes of novel stem cell‐based tissue reconstruction have been reported, and curative effects were accomplished in some other cases. However, in some cases, the expected therapeutic effects were not achieved depending on the type of stem cells used and the donor background.^35^, ^36^ Mesenchymal stem cells are widely used for regenerative therapy because they can be obtained from various tissues across the whole body, including adipose tissue. However, a meta‐analysis assessing the effectiveness of stem cell therapy for type 1 diabetes (T1DM) and type 2 diabetes (T2DM) showed no significant improvements by mesenchymal stem cells (MSC) in HbA1c and C‐peptide levels, which are indicators of blood glucose levels.^37^ This may be related to the deteriorated stem cell quality due to the degeneration of nuclear membranes and mitochondria, in mesenchymal stem cells derived from patients with diabetes, as revealed in our study. Moving forward, we hope to characterize mesenchymal stem cells from patients with diabetes in detail to identify a method to establish stem cells suitable for transplantation.

In this study, we successfully found structural differences in the nuclear membrane and decreased mitochondrial enzyme activity in the adipose‐derived mesenchymal cells from patients with type II diabetes. These results demonstrated the existence of type II diabetes‐associated changes in mesenchymal stem cells for regenerative therapy. Our findings are also useful for stem cell quality control in regenerative therapy for diabetic patients using autologous stem cells.

## CONFLICTS OF INTEREST

The authors confirm that there are no conflicts of interest.

## AUTHOR CONTRIBUTIONS

**Michiko Horiguchi:** Conceptualization (lead); Data curation (lead); Formal analysis (lead); Funding acquisition (lead); Investigation (lead); Methodology (lead); Project administration (lead); Resources (lead); Software (lead); Supervision (lead); Validation (lead); Visualization (lead); Writing‐original draft (lead); Writing‐review & editing (lead). **Shinichi Hata:** Data curation (supporting); Writing‐review & editing (supporting). **Yuya Tsurudome:** Data curation (supporting); Writing‐review & editing (supporting). **Kentaro Ushijima:** Data curation (supporting); Formal analysis (supporting); Investigation (supporting); Writing‐review & editing (supporting).

## Data Availability

The data that support the findings of this study are available from the corresponding author upon reasonable request.
